# Vibrational spectroscopy as a probe of heterogeneities within geochemical thin films on macro, micro, and nanoscales†

**DOI:** 10.1039/d3ra05179j

**Published:** 2023-10-02

**Authors:** Deborah Kim, Samantha Townsley, Vicki H. Grassian

**Affiliations:** a Department of Chemistry and Biochemistry, University of California San Diego La Jolla CA 92093 USA vhgrassian@ucsd.edu

## Abstract

Minerals play a critical role in the chemistry occurring along the interface of different environmental systems, including the atmosphere/geosphere and hydrosphere/geosphere. In the past few decades, vibrational spectroscopy has been used as a probe for studying interfacial geochemistry. Here, we compare four different vibrational methods for probing physical and chemical features across different mineral samples and length scales, from the macroscale to nanoscale. These methods include Attenuated Total Reflection – Fourier Transform Infrared (ATR-FTIR), Optical Photothermal Infrared (O-PTIR), Atomic Force Microscopy-Infrared (AFM-IR) and micro-Raman spectroscopy. The emergence of these micro-spectroscopic probes has offered new insights into heterogeneities within geochemical thin films and particles. These developments represent an important step forward for analyzing environmental interfaces and thin films as often these are assumed to be physically and chemically homogeneous. By comparing and integrating data across these measurement techniques, new insights into sample differences and heterogeneities can be gained. For example, interrogation of the various mineral samples at smaller length scales is shown to be particularly informative in highlighting unique chemical environments, including for chemically complex, multicomponent samples such as Arizona Test Dust (AZTD), as well as differences due to crystal orientation.

## Abstract 2

Geochemical thin films of different minerals and mineral samples were analyzed using several vibrational probes including microspectroscopic photothermal IR probes to uncover heterogeneity in these samples on micro and nanoscales.

## Graphical abstract

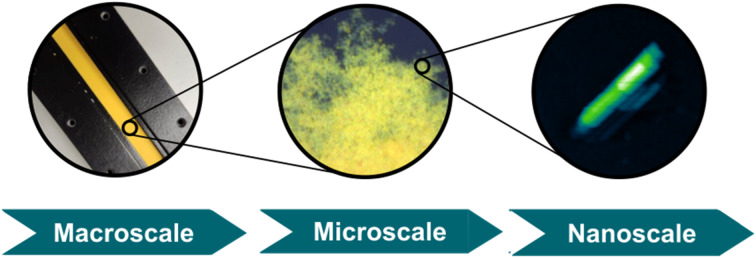

## Introduction

Minerals play a critical role along the interface of different environmental systems. Interfacial geochemistry, which occurs at the intersection of the atmosphere/hydrosphere and geosphere, plays an important role in air quality, water quality, elemental cycling, climate, soil contamination, food production and even the fate of environmental deoxyribonucleic acid (eDNA).^1–4^ Minerals in contact with groundwater and other water systems play a major role in the transport of contaminants in the environment. The complexity prevalent in environmental samples and interfaces, in particular, is largely due to the myriad of compounds and chemical species as well as the heterogeneity found in these samples. For geochemical interfaces, whether in mineral dust aerosols that are transported in the atmosphere or minerals that are in contact with groundwater, the mineralogy-specific, surface facet-dependent chemistry is important to unravel.^5,6^

Mineral dust aerosol drives a great deal of interfacial geochemistry in the atmosphere, although volcanic eruptions and volcanic ash also play a role. Mineral dust aerosols are highly complex and are composed of a myriad of different mineral oxides and oxyhydroxides, as some of the most well-studied components.^7–11^ Iron oxides and oxyhydroxides, such as hematite and goethite in particular, are some of the most prevalent due to the natural abundance of iron.^12–14^ Iron-containing minerals are an important component of mineral dust aerosols as iron plays an important role in different biogeochemical cycles.^14–16^ Carbonate minerals, including calcite, are naturally present in mineral dust aerosols but also in industrial buildings as well as in the ocean as coral.^7,11^ Clays, such as kaolinite and montmorillonite, represent an abundant class within mineral dust aerosols.^17^ Zeolites, crystalline aluminosilicates with porous structures, are found naturally and are also produced for use in environmental remediation.^2^

Furthermore, mineral dust aerosols undergo heterogenous reactions in the atmosphere with gas-phase nitrogen and sulfur oxides that result in the formation of sulfate or nitrate coatings that drive different atmospheric processes.^9,18,19^ Representative compounds include sodium sulfate, sodium nitrate and ammonium sulfate. These different species and coatings on the surface of mineral dust particles can impact the subsequent properties of dust.^18,20^ Surface coatings can also directly participate in cloud condensation nuclei activity or be a potential source for HONO production in the atmosphere in the case of nitrate coatings.^21–23^ In addition, surface nitrate and sulfate can also act as common adsorbates and can compete for sites on mineral surfaces.^24,25^

Vibrational spectroscopy can be used as a probe of different minerals and surface coatings by monitoring the different vibrational modes and unique chemical signatures from different functional groups. This allows for the identification of different chemical species and phases present as well as a way to distinguish some of the unique “local environments” due to hydration, complexation, different phases, and other chemical and physical heterogeneities within environmental systems.^26,27^ By monitoring the peak position, shape, and intensity of vibrational bands, molecular level insights can be gained. While a great deal of the current literature has used different vibrational methods, the recent development of micro-spectroscopic probes has been found to provide information on much smaller length scales.^24,28–35^ Besides local chemical environments, differences in spectra due to heterogeneities of film thickness and crystal orientation with respect to laser polarization also effect peak intensities and frequencies in the vibrational spectra.

Interrogating geochemical interfaces and thin films on micro and nanoscales can be particularly insightful when trying to understand the complexity of these interfaces. By utilizing these different vibrational spectroscopic probes, the goal is to provide insights into these samples on multiple length scales, *i.e.*, macroscale, microscale, and nanoscale, for different geochemical thin films that vary in their complexity and heterogeneity. We have previously shown the utility and the new insights that can be gained when comparing measurements on the macroscale to the nanoscale in the interactions of organic matter, oxyanions and proteins with mineral surfaces.^24,28^ Here we focus on the heterogeneities inherent in geochemical samples and expand these studies using macro, micro and microscale probes. In particular, we present here an analysis of several oxides/oxyhydroxides, carbonates, nitrates, sulfates and aluminosilicate samples as well as a more complex, multi-component sample such as Arizona Test Dust (AZTD). These different vibrational methods used have different modes of operation, different principles of operation, and, in some cases, different selection rules. Furthermore, these spectra can be used as a library of information for the analysis, identification and characterization of the mineralogy present within different environmental samples.

## Materials and methods

### Sample preparation and sources of materials

Thin films of different minerals were prepared for analysis. Each thin film prepared for ATR-FTIR, AFM-IR, O-PTIR, and micro-Raman spectroscopic data collection and analysis originated from the same sample and same particle size fraction in order to minimize any additional variables that could affect these samples and their vibrational spectra. Oxide solids were suspended into Milli-Q water at a concentration of 5 mg mL^−1^ and then sonicated for two minutes to de-aggregate the sample. This process resulted in a homogenous suspension. For carbonate, sulfate, and nitrate samples, the concentration of the stock solutions was 0.1 M. All compounds were purchased commercially, and a compiled list of the sources of these materials can be found in ESI Table S1.†

Once the solutions and suspensions for these different samples were made, a fixed volume of each solution was drop-cast onto a designated substrate and dried. This produced a thin, mineral particle film. While each of the films originated from the same stock solution, the amount of volume and drying time varied for each substrate and technique. For ATR-FTIR spectroscopic measurements, a 1 mL aliquot of the stock solution was deposited onto a cleaned, Amorphous Material Transmitting Infrared Radiation (AMTIR) crystal and allowed to air-dry for at least 5 hours. For AFM-IR and O-PTIR spectroscopic measurements, a 5 μL aliquot of the stock solution was drop-casted onto a cleaned silica wafer and calcium fluoride (CaF_2_) substrate, respectively, and allowed to air-dry for at least 3 hours. Both substrates were chosen specifically for each respective technique due to the absence of spectral interference. For single particle AFM images, the same procedure discussed above was repeated but with a stock solution that was diluted five-fold. This was done to prepare thinner films to reduce the presence of aggregates. Upon drying, each of the films were analyzed.

### ATR-FTIR spectroscopy: Nicolet iS10 FTIR spectrometer

ATR-FTIR spectroscopy is a macroscopic measurement technique that is based upon the principles of total internal reflection. An incoming broadband infrared light source is reflected internally at the interface between an optically dense medium, the ATR crystal, and an optically rare medium, the sample, where the angle of incidence exceeds the critical angle. A peak is detected when the wavelength of light is in resonance with a vibrational mode, similar to transmission absorption infrared spectroscopy.^36–38^ The absorbance, *A*, can be written as a modified Beer's law as shown in eqn (1),^40^

1 *A* = *εNd*_e_*c*

where *ε* refers to the molar absorption coefficient, which is a function of wavenumber, and *N* refers to the number of internal reflections at the interface of the ATR crystal and sample. *d*_e_ refers to the effective depth of penetration, and *c* is the concentration. The depth of penetration, *d*_p_, of this IR beam into the sample can be expressed by eqn (2):^36^2
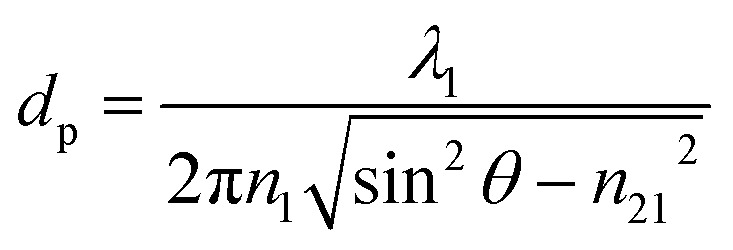
Here, *λ*_1_ refers to the wavelength of light divided by the refractive index of the ATR crystal and *θ* represents the angle of incidence of the IR beam. *n*_21_ is representative of the ratio of the refractive indices of an optically dense medium (ATR crystal, *n*_2_) to the optically rare medium, (sample, *n*_1_).^36,39^

For ATR-FTIR spectroscopic measurements, a 500 μL Teflon-coated horizontal flow cell, housing an AMTIR crystal (Pike Technologies) with a refractive index of 2.5, with an angle of incidence at 45°, was used. While the capped flow cell can hold 500 μL in liquid volume, when open, the trough of the cell can hold up to 1 mL in liquid volume. Once the thin films were prepared and dried on the AMTIR crystal, each IR spectrum was recorded at a resolution of 4 cm^−1^ and were averaged over 100 scans in the spectral range from 725 to 4000 cm^−1^. For these measurements, a commercial instrument, a Nicolet iS10 FTIR spectrometer (Thermo Fischer Scientific), equipped with an MCT-A detector was used. Each spectrum was processed using the OMNIC software and plotted with Origin. There were no corrections applied to the data to account for the wavelength dependent probe depth and anomalous dispersion of the refractive index which can cause band shifts of *ca.* 2 to 10 cm^−1^.

### O-PTIR and micro-Raman spectroscopy: mIRage-Raman

O-PTIR and micro-Raman spectroscopic data were collected using a mIRage infrared + Raman microscope system (Photothermal Spectroscopy Corp., Santa Barbara, CA) with a continuous wave, 532 nm laser. O-PTIR spectroscopy is a technique based upon a photothermal response caused by infrared absorption of a sample. Unlike traditional IR spectroscopy, this new technique is an indirect method, whereby the signal is a result of a change in the refractive index. By using a visible light source that is collinear to the IR laser beam as the probe, an O-PTIR signal is generated as a response to the absorption of an infrared photon that is resonant with a specific vibrational mode. This is followed by a warming of the sample and a thermal expansion which produces a thermal response that is detected by a change in the refractive index. The modulated probe beam is reflected back to the detector and can be expressed as Δ*P*_pr_, since it is a change in the refractive index of the probe. This change is representative of the O-PTIR signal, which is linearly proportional to the IR absorbance of the sample, as shown in eqn (3):^41^3
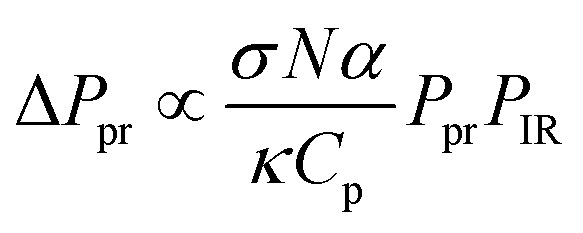


The first few terms in eqn (3) describe the physical property of the sample, where *σ* is the absorption cross-section which is a function of wavenumber, *N* is the number density, *κ* is the heat conductivity, *C*_p_ is the heat capacity and *α* is the thermo-optic coefficient. The probe and infrared laser powers are represented by *P*_pr_ and *P*_IR_, respectively.^41–44^ In addition, the Raman scattering signal is generated simultaneously, as the scattered photons from this modulated signal are then diverted to a Raman spectrometer^42^ consisting of a monochromator and charge-coupled device (CCD) detector. These simultaneous measurements (O-PTIR and Raman) provide complementary spectroscopic features that are useful for mineral identification. Most importantly, this instrument has a spatial resolution that is determined by its visible light probe, which is on the order of 0.6 μm.^45,46^

For the O-PTIR spectroscopic data collection, optical images of samples are first collected with two objectives: a 10×, low magnification, visible objective lens and a 40×, high magnification reflective Cassegrain objective lens. It should be noted that the O-PTIR spectra are collected with the 40× reflective Cassegrain objective, while the 10× visible objective is used to provide a lower resolution optical image of the sample. The system is also equipped with two mid-IR tunable lasers: a quantum cascade laser (QCL) and an optical parametric oscillator (OPO) laser. In theses experiments s-polarized light is used. The spectral range for the QCL laser is 755 to 1855 cm^−1^ and the spectral range for the OPO laser is 2600 to 3600 cm^−1^. In addition to the IR spectra, the mIRage system also yields Raman data in the 400 to 4000 cm^−1^ spectral range from the 532 nm laser. Once samples are prepared onto CaF_2_ substrates, they are placed in the sample compartment of the instrument for analysis. Settings for probe and IR laser power are determined by optimizing the photothermal signal. The probe power ranges anywhere from 0.5% to 19% while the IR laser power ranges from 20% to 100%. For micro-Raman spectral measurements, the most optimal probe power and integration time are determined by the settings that give the highest signal-to-noise ratio. The probe power for Raman ranges from 0.2% to 19% while the integration time ranged from 1 to 10 seconds. All spectra are collected at a resolution of 1 cm^−1^. After data collection, spectra are baseline corrected using the Photothermal PTIR Studio software and plotted with Origin.

Micro-Raman spectroscopy measures the amount of scattered light from a sample and is often used for mineral identification.^47,48^ While infrared allowed vibrational modes are a result of a change in dipole, Raman scattering occurs for vibrational modes that result in a change in polarizability, *α*, which leads to an induced dipole, as shown in eqn (4),^49,50^

4 *μ*_ind_ = *αε*,

where *ε* refers to the electric field that is driven by light and the resulting induced dipole, *μ*_ind_.

### AFM-IR spectroscopy: NanoIR2

AFM-IR spectroscopy is a hybrid technique that combines the nanoscale spatial resolution of AFM with the chemical analysis capability of IR spectroscopy by using the cantilever tip as the detector for IR absorbance. High spatial resolution on the scale of the tip diameter can be achieved. Like O-PTIR spectroscopy, AFM-IR spectroscopy is also a photothermal response to absorption of infrared light.^51^ This localized thermal expansion is known as photothermal induced resonance (PTIR), where the sample expansion is detected mechanically through a device that is sensitive to changes in force and displacement, such as an AFM cantilever tip, which provides nanoscale spatial resolution. Thus, AFM-IR spectroscopy to be used for chemical characterization of samples as it proportionally relates the thermal expansion to the cantilever deflection. This transduced signal can be represented by eqn (5):^51^

5 *S*_*n*_(*ω*_*n*,_*σ*) = *H*_m_*H*_AFM_*H*_opt_*H*_th_*σκ*(*σ*)

*H*_m_ refers to the mechanical contribution from the sample, *H*_AFM_ accounts for the cantilever contribution from the deflection, *H*_opt_ is the optical contribution and *H*_th_ is the thermal contribution. This equation represents the photo thermal infrared response as a function of frequency, *ω*_*n*_, and wavelength, *σ*, where the changes in the detected signal are largely dependent on the response of the sample to the wavelength of light illuminated onto the sample.^51–55^

For AFM-IR images and spectra, a nanoIR2 microscopy system (Bruker, Anasys – Santa Barbara, CA) is used. Similar to the mIRage-Raman, the system is equipped with a QCL laser, with a spectral range from 800 to 1800 cm^−1^. It should be noted that the system is also equipped with a pulsed, tunable optical parametric oscillator (OPO) laser that has a spectral range of 850 to 2000 cm^−1^ and 2235 to 3600 cm^−1^. While this laser offers a range that can provide chemical information regarding the C–H, N–H, and O–H region, it was not used for this study because the spectra can only be collected in contact mode, which, due to the roughness of these thin films, was extremely difficult for image collection. Therefore, tapping mode had to be used in order to collect clear AFM height images that corresponded with the photothermal infrared spectra. Regardless, the QCL region provides high quality spectra in the fingerprint region. Once samples are prepared and dried onto the silica wafer, the infrared spectra and images are collected primarily in Tapping AFM-IR mode, using a gold-coated silicon nitride probe (Bruker), with tip radii of ∼30 nm, a resonant frequency of 75 ± 15 kHz and a spring constant of 1 to 7 N m^−1^. The scan rate for imaging and mapping varies depending on the roughness of each thin film but within the range of 0.1 to 0.8 Hz. All images are collected at a resolution of 512 pixels and all spectra are collected at a resolution of 2 cm^−1^ with s-polarized light. After data collection, AFM images and infrared spectral maps are processed using the Gwyddion software and plotted in Origin.

## Results and discussion

### Vibrational spectroscopy of oxides and oxyhydroxides

**Fig. 1:**
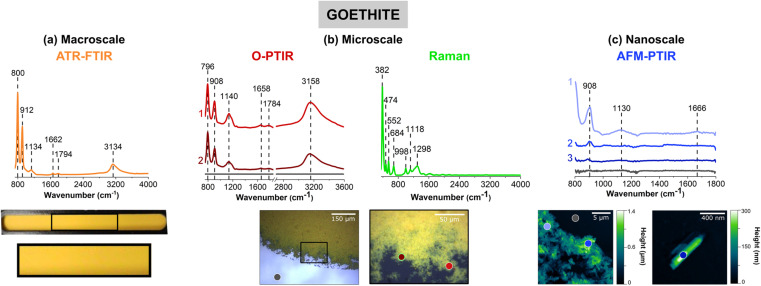
Comparison of (a) macroscale, (b) microscale, and (c) nanoscale spectral analysis for goethite (α-FeOOH), collected with ATR-FTIR spectroscopy, O-PTIR + Raman spectroscopy and AFM-IR spectroscopy, respectively. Point spectra collected using O-PTIR, Raman, and AFM-IR spectroscopy are shown. In (a), a picture of the thin film on the ATR crystal is shown and the portion in a black box is expanded further. Optical images of a goethite thin film are presented in (b) at low magnification (10×) and high magnification (40×) of the region specified by the black box. AFM 2D-height images of the goethite thin film as well as a single cluster of goethite nanoparticles are shown in (c). For microscale and nanoscale analysis, the colored point spectra correspond to the colored dots on the image. The colored dot with the green outline indicates the region in which the Raman spectrum was taken. Spectra taken on the substrate, for both O-PTIR and AFM-IR spectroscopy, are shown by the dark gray dot and the gray spectrum.

Goethite, α-FeOOH, an iron-containing oxyhydroxide, was chosen as a representative mineral due to its prevalence in the environment and its importance in iron cycling. Fig. 1 compares the four different vibrational spectroscopic probes used to analyze goethite thin films over three different length scales.

Thin films prepared for each sample are shown below the different spectra collected. Fig. 1a shows a macroscopic image of a goethite thin film on the ATR crystal, where the film appears to be, by visual inspection, homogenous and yellow/orange in color. However, the images in Fig. 1b, taken with the mIRage-Raman system, show there are regions of different thickness and smaller clusters and particles on the micrometer length scale at the edge of the film. While the film appears to be somewhat homogenous toward the center of the film in the 10× optical image, the 40× optical image shows greater variability with thicker and thinner regions. For the nanoIR2 system (Fig. 1c), individual goethite clusters can be resolved, and the highest spatial resolution image shown gives a more detailed image of single particles within the thin film. This progression of images from macroscale, microscale, and nanoscale imaging highlights the physical heterogeneity of the goethite thin film. This heterogeneity is subsequently explored and developed through combined spectral analysis as discussed below.

The spectral range for each instrument is slightly different due to the different detectors and/or light sources as already noted in the Materials and methods section. For ATR-FTIR, O-PTIR, Raman, and AFM-IR spectroscopies, the spectra regions correspond to 725 to 4000 cm^−1^, 755 to 3600 cm^−1^, 400 to 4000 cm^−1^, and 800 to 1800 cm^−1^, respectively. When the infrared peak frequencies are compared for the three infrared methods, ATR-FTIR, O-PTIR, and AFM-IR, they are typically within 10 cm^−1^ for some of the sharper, narrower peaks whereas for some of the broader peaks, the differences can be as much as 40 cm^−1^.

Goethite has two distinct peaks below 1000 cm^−1^, one near 795 cm^−1^ and the other near 910 cm^−1^. Both of these peaks can be observed in the spectra for all three different measurement modes. These two peaks are assigned to the in-plane and out-of-plane bending modes of the OH groups, respectively.^12,13^ The presence of the peak near 795 cm^−1^ in the AFM-IR data is not as clear because of the nanoIR2 cutoff at 800 cm^−1^. The broad O–H stretching mode is seen in both the ATR-FTIR and O-PTIR spectra, as a broad band with a peak frequency maximum between 3135 and 3160 cm^−1^.^12,13^

Spectral shifts are observed between the different methods which is a result of several factors, including the use of different substrates with different specular reflectance.^56^ Differences in spectral intensity with the same technique but for different spots on the same sample attributed to variation in the sample thickness. For the two laser-based methods, O-PTIR and AFM-IR, the orientation of the particles within the thin film will be a factor.^54,57^ Since the laser light is polarized, single particles of different orientation can give rise to different spectra, the exact differences depend on crystal symmetry heterogeneity within the thin film. This typically manifests in different spectral shifts and/or intensity differences.

The novelty of micro-spectroscopic studies lies in the ability to probe smaller length scales since the spatial resolution is no longer determined by the wavelength of light used, which in the infrared is on the order of a several microns. With the mIRage-Raman, as shown in Fig. 1b, point spectra can be resolved on the order of sub-microns. The simultaneous acquisition of micro-Raman spectra provides information about Raman active modes are used for mineral chemical identification, especially for more complex samples. Fig. 1b shows a micro-Raman spectrum of goethite with several identified at 382 cm^−1^, 474 cm^−1^ and 552 cm^−1^, and 684 cm^−1^, which are assigned to Fe–O–Fe stretching modes, Fe–OH asymmetric stretching modes, and Fe–O symmetric stretching modes, respectively.^58^ Peak positions and assignments for the micro-Raman spectrum of goethite are also given in Table 1. Most importantly, micro-Raman spectra provide low frequency vibrations below 700 cm^−1^ that are particularly useful for mineral identification.

**Table 1:** Vibrational mode assignments for goethite, sodium nitrate and kaolinite and frequencies measured from ATR-FTIR, O-PTIR, AFM-IR and micro-Raman spectroscopies

Sample	Wavenumber (cm^−1^)			Assignment	Wavenumber (cm^−1^)	Assignment
	ATR-FTIR	O-PTIR	AFM-IR		Raman	
Goethite^10,11,51,52,55,56^	800	796	—	In-plane, O–H bend	382	Fe–O–Fe, O–H symmetric stretch
	912	908	908	Out-of-plane, O–H bend	474, 552	Fe–OH asymmetric stretch
	1134	1140	1130	O–H bend	684	Fe–O symmetric stretch
	1662	1658	1666	H–O–H bend	—	—
	1794	1784	—	Goethite overtone bend	—	—
	3134	3158	—	O–H stretch	—	—
Sodium nitrate^57–59^	834	834	834	υ_2_, out-of- plane deformation	720	υ_4_, in-plane bend
	1348, 1440	1350, 1390, 1444	1342, 1352, 1374, 1396	υ_3_, asymmetric stretch	1070	υ_1_, symmetric stretch
	—	—	—	—	1384	υ_3_, asymmetric stretch
Kaolinite^60–64^	796, 916, 1026, 1114	798, 924, 1018, 1118	920, 998, 1034, 1056, 1080, 1120	Al–OH stretch, O–H bend	3624, 3700	O–H stretch
	1026	924, 1018, 1118	998, 1034, 1056, 1080, 1120	In-plane Si–O stretch		
	3618, 3651, 3670, 3696	—	—	O–H stretch	—	—

Point spectra obtained on particles or clusters on smaller length scales with AFM-IR spectroscopy are shown in Fig. 1c. The two images show micron and nanometer length scales that provide information that is below the range of the mIRage-Raman system. Even a single goethite nanocluster can be spatially resolved and a vibrational spectrum collected. The vibrational spectra collected with the AFM-IR instrument is for a *ca.* 30 nm diameter region.

Measurements were also made on several oxide samples. Spectra for corundum (α-Al_2_O_3_), anatase (TiO_2_) and amorphous silicon dioxide (SiO_2_) are shown in Fig. S1 in ESI.† The differences within the O-PTIR spectra can be attributed to a few factors. These thin films, composed of a crystalline powder of single particles, will be oriented with respect to the polarization of the infrared beam for the laser-based techniques. Combined with the local thickness variations within these films, small spectral shifts and differences and intensities are observed as already noted for AFM-IR data.^54,57,59^ The different mineral films have different degrees of roughness as some thin films dry more uniformly than others. For samples like titanium dioxide, the micro-Raman spectrum can be extremely helpful in identifying which phase is present as different phases of titanium dioxide – rutile, anatase and brookite – all have different low frequency modes.^60,61^ In the case of amorphous SiO_2_, there was considerable fluorescence observed from the sample (see Fig. S1 in ESI†). Specific IR and Raman peak assignments for these compounds are listed in Tables S2 and S3, respectively, in ESI.†

### Vibrational spectroscopy of nitrates, carbonates, and sulfates

**Fig. 2:**
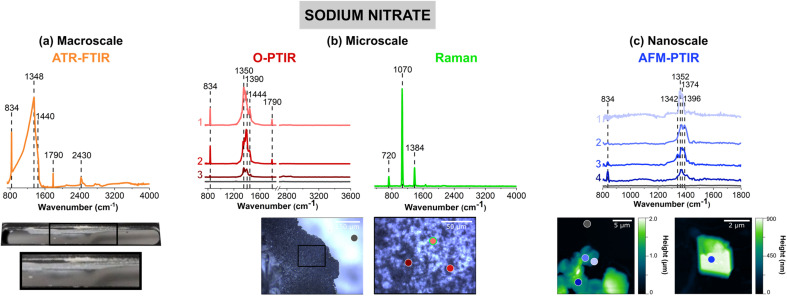
Comparison of (a) macroscale, (b) microscale, and (c) nanoscale spectral analysis for sodium nitrate thin films, collected with ATR-FTIR spectroscopy, O-PTIR + Raman spectroscopy and AFM-IR spectroscopy, respectively. Point spectra collected using O-PTIR, Raman, and AFM-IR spectroscopy are shown. In (a), a picture of the thin film on the ATR crystal is shown and the portion in a black box is expanded further. Optical images of a sodium nitrate thin film are presented in (b) at low magnification (10×) and high magnification (40×) of the region specified by the black box. AFM 2D-height images of the sodium nitrate thin film as well as a sodium nitrate cluster are shown in (c). For microscale and nanoscale analysis, the colored point spectra correspond to the colored dots on the image. The colored dot with the green outline indicates the region in which the Raman spectrum was taken. Spectra taken on the substrate, for both O-PTIR and AFM-IR spectroscopy, are shown by the dark gray dot and the gray spectrum.

Fig. 2 shows vibrational spectra collected of sodium nitrate thin films and individual particles. Sodium nitrate has distinct vibrational peaks due to the symmetric and asymmetric stretching and bending modes of nitrate anion. For infrared modes, ATR-FTIR, O-PTIR, and AFM-IR spectroscopy show the most intense bands associated with the asymmetric stretch of the nitrate anion in the region between 1342 to 1444 cm^−1^ (*vide infra*).^62^

The image of a sodium nitrate thin film on the ATR crystal (Fig. 2a) shows that there are aggregates and irregular-shaped precipitates along the side of the crystal. The 40× optical image shows patches of sodium nitrates clusters, as seen by the different reflective regions within the thin film. Utilizing AFM, Fig. 2c offers a closer look at the edge of a sodium nitrate thin film as well as individual particles. The AFM 2D height images show that these sodium nitrate particles are irregular-shaped with varying heights. The 20 μm × 20 μm image of the thin film shows that the thin film is oriented in a way where the nitrate clusters are packed together to form taller, 2 μm films. However, from the 5 μm × 5 μm image shown on the right (Fig. 2c), the nanoscale features indicate the variance in height within a single particle. Each of these features do not appear to be nano-sized, but with the nanoIR2 system, the heterogeneity within these clusters can be probed effectively.

For sodium nitrate, there are important variations that should be noted when comparing the macroscopic nitrate spectra across scales and with different techniques. Most notable are the differences in the spectral region from 1340 to 1445 cm^−1^, which can be clearly observed in terms of peak frequency, peak shape, and relative intensity. The nitrate anion has a *D*_3h_ symmetry and has four vibrational modes – υ_1_, υ_2_, υ_3_, and υ_4_ – which represent the symmetric stretch, out-of-plane deformation, asymmetric stretch, and in-plane bend, respectively. The υ_3_ nitrate asymmetric stretching mode is the most prominent peak and it is also doubly degenerate, and its vibrational mode frequency shifts the most in different local environment and changes in symmetry due to hydration state, crystallinity and coordinating cations. The loss of symmetry is evident by the υ_3_ nitrate asymmetric stretching mode splits into two modes: a broad band from 1342 cm^−1^ to 1444 cm^−1^ and a sharper band at 834 cm^−1^. This splitting is highly dependent on the reduction in symmetry and distortion of the nitrate anion away from *D*_3h_ symmetry, which depends on the hydration, crystallinity, and coordination environments. For example, even in the hydration sphere of bulk aqueous systems, the υ_1_ mode, albeit quite weak, becomes infrared active.^63–66^ In addition, the spectra of highly crystalline compounds of high symmetry will depend on light polarization.^54^ The effect of polarized light on infrared spectra of crystalline nitrates showed the changes in nitrate absorptions using different light polarizations.^67^ Additional for crystalline anisotropic materials variations and their interactions with polarized light from microspectroscopic probes have showed variations in spectral intensities and peak positions.^68^

For the ATR-FTIR spectrum shown in Fig. 2a, there is a very intense, broad peak centered around 1384 cm^−1^ associated with nitrate asymmetric stretch. Therefore, by increasing the spatial resolution more defined peaks that contribute to the breadth of this intense feature may be resolved. In addition, to the asymmetric stretch, there is a sharper band at 834 cm^−1^ due to the out-of-plane nitrate bend. O-PTIR spectroscopy can offer more insight into differences in the local nitrate environment. For example, there are differences in the peak maximum observed for different parts of the film, in the spectrum labeled as 1 (pink) in Fig. 2b the peak maximum observed at 1350 cm^−1^ whereas the spectrum labeled 2 (red) the peak maximum is shifted to higher frequencies 1390 cm^−1^. Additionally, the spectrum labeled 3 (dark red) shows to have a doublet within the same region. For these three spectra, there is also a small shoulder *ca.* 1445 cm^−1^. The AFM-IR spectra also show variability. In particular, spectrum 1 (light violet) has a major peak at 1396 cm^−1^ and spectrum 2 (violet) exhibits a doublet at 1368 cm^−1^ and 1396 cm^−1^. The differences in peak intensity can also be observed, with Spectrum 3 (blue) in Fig. 2b and Spectrum 4 (dark blue) in Fig. 2c, having the lowest peak intensity. While the differences in intensity or minor peak shifts can be attributed to changes in substrates and sample thickness, the major differences in the peak positions are more likely due to changes that occur in the nitrate symmetry – due to differences in crystallinity and the presence of small amounts of water due to incomplete drying.

Although differences in the infrared spectra were evident of different nitrate local environments, the micro-Raman spectra were consistent across all spectra, and a single spectrum is shown in Fig. 2b. There are three distinct Raman peaks at 720 cm^−1^, 1070 cm^−1^, and 1384 cm^−1^ which are representative of the NO_3_^−^ υ_4_, in-plane bend, υ_1_, the symmetric stretch, and υ_3_, the asymmetric stretch, respectively.

Other minerals investigated include calcite, sodium sulfate, and ammonium sulfate. Calcite has distinct peaks at 874 cm^−1^, 1400 to 1514 cm^−1^, and 1796 cm^−1^ (Table S4 in ESI†). These bands represent the out-of-plane bending, asymmetric stretching mode, and a combination band, of the carbonate ion, respectively.^69,70^ The variation in the intensity and small spectral shifts for calcium carbonate can be attributed to the differences in thickness. These features are representative for different carbonate phases but with slightly different frequencies. In addition, nanoscale features for calcium carbonate clusters can be resolved. These clusters are small and are seen aggregating when cast into a thin film. The micro-Raman spectrum of calcite shows a combination of bending and stretching modes of carbonate at 714 cm^−1^, 1090 cm^−1^ and 1440 cm^−1^, respectively. The 1758 cm^−1^ peak in the Raman spectrum is most likely attributed to a combination band.^71^ The Raman peak assignments are provided in ESI Table S5.†

Like nitrates, sulfates are ubiquitous oxyanions in the environment. The vibrational frequencies for the sulfate anion also depend on hydration state as well as the local environment. For the unperturbed anion, sulfate is of tetrahedral symmetry.^72–74^ These shifts for both sodium and ammonium sulfate that are observed in the spectra are listed in Table S4 (ESI).† For ammonium sulfate, similarities between the υ_3_ SO_4_^2−^ stretching mode in the 1080 to 1170 cm^−1^ region and the υ_4_, NH_4_^+^ stretching mode in the 1412 to 1420 cm^−1^ region are seen between different scales.^72,74–76^ However, there are notable differences in the intensity seen in the point spectra. Specific IR and Raman peak assignments for these minerals are listed in Tables S4 and S5, respectively, in ESI.†

### Vibrational spectroscopy of aluminosilicates: clays and zeolites

**Fig. 3:**
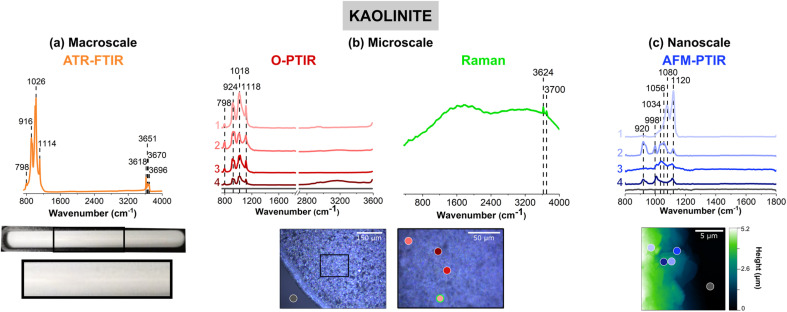
Comparison of (a) macroscale, (b) microscale, and (c) nanoscale spectral analysis forkaolinite thin films, collected with ATR-FTIR spectroscopy, O-PTIR + Raman spectroscopy and AFM-IR spectroscopy, respectively. Point spectra collected using O-PTIR, Raman, and AFM-IR spectroscopy are shown. In (a), a picture of the thin film on the ATR crystal is shown and the portion in a black box is expanded further. Optical images of a zeolite thin film are presented in (b) at low magnification (10×) and high magnification (40×) of the region specified by the black box. AFM 2D-height images of the kaolinite thin film are shown in (c). For microscale and nanoscale analysis, the colored point spectra correspond to the colored dots on the image. The colored dot with the green outline indicates the region in which the Raman spectrum was taken. Spectra taken on the substrate, for both O-PTIR and AFM-IR spectroscopy, are shown by the dark gray dot and the gray spectrum.

Fig. 3 shows vibrational data for kaolinite, a clay mineral, which is known to contain different stoichiometric ratios of alumina and silica derivatives.^77^Fig. 3a shows a very lightly colored beige film that uniformly adheres to the ATR crystal. Upon closer inspection of the film with the mIRage-Raman, small features that vary in size and reflectivity are seen in the low magnification, 10× image. The AFM height image shows that this film is thick, at 5 μm in height. Unlike goethite and sodium nitrate, for kaolinite there were not obvious single particles but instead particles were associated with the film.

An ATR-FTIR spectrum of kaolinite is shown in orange in Fig. 3a. The spectrum shows an intense triplet at 916 cm^−1^, 1026 cm^−1^, and 1114 cm^−1^, as well as peaks in the O–H stretching regions from 3620 to 3700 cm^−1^. This region is indicative of aluminosilicate derivatives in clays as well as zeolite or mineral oxides such as aluminum oxide and silicon dioxide, as shown in Table S2 in ESI.†^77–80^ It should be noted that the relative intensities of the three peaks is that the 1026 cm^−1^ is highest in intensity, followed by the 916 cm^−1^ peak, and then the 1114 cm^−1^ peak.

The heterogeneity of the kaolinite sample is evident for the data collected with the mIRage-Raman system. In particular, the ratio of the three main peaks differs within each of the O-PTIR spectra. For example, in Fig. 2b, the spectra labeled as 1 (light pink), 3 (red) and (dark red), show ratios of the three peaks resembles that of the ATR-FTIR spectrum in Fig. 2a qualitatively but quantitatively are different. In addition, the peak at 1018 cm^−1^ is broader in 3 (red) than 1 (light pink) whereas the peak at 924 cm^−1^ is sharpest for 1 (light pink). Furthermore, the spectrum labeled 2 (pink), has a different ratio, where the 924 cm^−1^ band is the strongest, followed by the 1018 cm^−1^ and 1118 cm^−1^. Lastly, for Spectrum 2, there is a more intense 798 cm^−1^ peak, which may be indicative of the Al–O Al bending mode.^81^ This peak is also as a minor contribution in the ATR-FTIR spectrum in Fig. 2a. From this microscale spectral analysis, evidence for heterogeneity is uncovered by the differences in intensity of the point spectra as well as differences in the ratios.

With AFM-IR spectroscopy, greater insights into local heterogeneities can be explored. Four spectra collected on different locations of the 20 μm × 20 μm region is shown in Fig. 3c. Significant differences in these spectra are important to note. Fig. 3c, the spectrum labeled 1 (light violet), has a prominent doublet at 1080 cm^−1^ and a more intense 1120 cm^−1^. Unlike that shown for 2 (violet) and 4 (dark blue), where the 920 cm^−1^ peak is absent, suggesting an absence of alumina derivatives in that region. In Fig. 3c, the spectrum labeled 2 (violet) is also particularly interesting, as it has a strong 920 cm^−1^ bending mode, along with sharper peaks at 998 cm^−1^ and 1120 cm^−1^ but a broad peak centered at *ca.* 1070 cm^−1^. This may suggest a strong presence of silica derivatives coupled to alumina and potentially other phases. Lastly, the spectrum labeled 3 (blue) is low in spectral intensity and has a broad peak from *ca.* 1000 cm^−1^ to 1120 cm^−1^. This region most likely is comprised of several silica derivatives, as it appears to be more poorly resolved compared to the other spectra. Overall, the nanoscale spectra provide the greatest amount of information on the heterogeneity of the sample. The varying stoichiometric ratios of silica and alumina derivatives are expected, as clay minerals such as kaolinite are complex and made up of tetrahedral and octahedral sheets of such compounds.

The micro-Raman spectrum of kaolinite shows high background signal indicative of fluorescence from the sample and two distinct features on top of that attributed to the inner and outer hydroxyl groups, at 3624 cm^−1^ and 3700 cm^−1^, respectively, as seen in the ATR-FTIR spectrum. The positions of these hydroxyl groups refer to where they are situated along the inner and outer planes of a kaolinite unit cell. Kaolinite is known to have two other outer hydroxyl groups that are not clearly resolved by the Raman spectrum shown, which may be due to the presence of other aluminosilicates that are present.^82,83^ The fluorescence observed in the micro-Raman spectrum of kaolinite resembles the spectra of montmorillonite and Arizona Test Dust, as can be seen in Fig. S3 and S4, respectively, in ESI.† Although fluorescence is commonly observed in such samples, the reasons for this vary. Ritz *et al.* suggests that fluorescence could occur due: (1) presence of organic matter, (2) presence of iron embedded into the clay mineral structure, and (3) even settings for Raman spectral data collection.^84–87^ Kaolinite is not known to contain high levels of cations such as iron or organic matter. However, both Ritz *et al.* and Frost *et al.* both suggest that fluorescence for clay minerals can be heavily dependent on its sources as well as the wavelength of light used for analysis.^77,84,87^ For example, it appears that the 1064 nm laser is preferred over the 532 nm laser for SWy-2 type of montmorillonite, which exhibits fluorescence, consistent with what is seen in Fig. S3 (ESI).† This information suggests that the fluorescence observed in this study is due to the presence of cations embedded in the mineral structure. For these samples, the zeolite sample is the only sample that showed a high quality micro-Raman spectrum. The vibrational mode assignments for the micro-Raman spectrum are given in Table S7 in ESI.†

The physical and chemical behavior exhibited by kaolinite is similar to some of the other aluminosilicate clays, zeolites, and complex multi-component samples. Optical and AFM images as well as chemical spectra of montmorillonite and zeolite are presented in Fig. S3 (ESI).† A consistent observation seen between all three types of spectra collected is that these compounds all show broad ATR-FTIR spectrum. When probed on the microscale, small differences in the spectra can be resolved, whether it be in intensity differences, as clearly shown by the zeolite O-PTIR spectra, or in peak-to-peak ratio shifts, as shown in the montmorillonite O-PTIR spectra. These micro and nanoscale differences most likely are contributed to in part to different chemical environments since compounds in this class of minerals are more heterogenous and complex by nature than the other minerals discussed above although crystal orientation and anisotropic crystal materials can result in intensity variations, peak frequencies and the presence of specific peaks in the spectrum.^54,57,59,68^

### Vibrational spectroscopy of a complex, multi-component sample: Arizona test dust

**Fig. 4:**
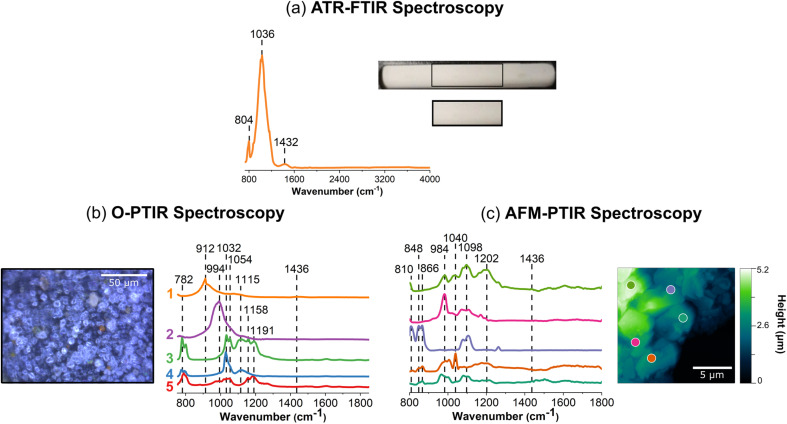
Comparison of infrared spectra collected for Arizona Test Dust (AZTD) from the three different instruments used in this study (a) Nicolet iS10 spectrometer (ATR-FTIR spectroscopy), (b) mIRage-Raman (O-PTIR spectroscopy) and (c) nanoIR2 (AFM-IR spectroscopy). A high magnification (40×) image of an AZTD thin film is shown in (c) while an AFM 2D height image is shown in (b). Selected point spectra are shown. Colored dots on the regions of the corresponding images match each of the colored spectra.

Mineral dust aerosols play an important role in the chemistry that occurs in the atmosphere as they are transported great distances and react with trace atmospheric gases.^32,88,89^ Arizona Test Dust has been used as a proxy for natural dust samples as it is easily accessible and well characterized in composition.^90^ Much of these aerosols originate from desert dust sources, and AZTD is a great model system to study as it contains a myriad of different elements, such as aluminosilicates, iron oxides, calcium carbonate, and potassium nitrate.^32,89,91^ In Fig. 4, the spectral heterogeneity of AZTD, a complex, multi-component sample, is compared using three different vibrational spectroscopic probes at macroscale, microscale, and nanoscale resolution.

Consistent with what has been observed for the aluminosilicates, AZTD shows similar features in the ATR-FTIR spectrum from 725 cm^−1^ to 1300 cm^−1^, as shown in Fig. 4a. The ATR-FTIR spectrum also indicates a peak at 1432 cm^−1^ which is attributed to the presence of carbonates (Table S4 in ESI†). O-PTIR spectra show variation in intensity and peak position. The high magnification optical image of AZTD depicts a highly heterogenous samples, with features that are more spherical or trapezoidal that vary in height, size, color, and shape, layered on top of one another. The nanoscale perspective by the AFM image provides quantitative information about the roughness of these samples, indicating that the height of the film can reach as high as 5 μm.

When comparing the spectra obtained between the mIRage-Raman and nanoIR2, there are clear variations observed in the 980 cm^−1^ to 1200 cm^−1^ region. In Fig. 4b, the spectra labeled 1 (orange) and 2 (purple) exhibit broad peaks at 912 cm^−1^ and 994 cm^−1^, respectively. These two regions suggest the presence of aluminosilicate derivatives. For example, spectra labeled 3 (green), 4 (blue), and 5 (red) all have a band *ca.* 780 cm^−1^, which is indicative of alumina as well as iron.^92^ Among these spectra, there is more variation in the *ca.* 1000 cm^−1^ to *ca.* 1200 cm^−1^ region, which suggests the varying stoichiometric ratios of alumina and silica, as well as the presence of some sulfates, which are known to be a component of AZTD.^91,92^ Broader spectral features for the O-PTIR spectra are attributed to the difference in spatial resolution, being able to distinguish between various minerals and components that make up this complex sample.

Fig. 4c provides additional evidence of the different species within AZTD. The five spectra in Fig. 4c are clearly distinct from one another. The spectra labeled 3 (purple), 4 (orange), and 5 (teal) show bands below 866 cm^−1^. In this region, we can expect alumina derivatives as well as carbonate derivates, as indicated by aluminum oxide and calcium carbonate in Tables S2 and S4 (ESI),† respectively. In contrast, the spectra labeled 1 (green) and 2 (pink) are absent of these peaks but are much richer in silica and sulfate derivatives. The data collected here can be used to better understand the composition of these complex mineral samples, along with other complementary to other techniques. For example, Cwiertny *et al.* has characterized AZTD using XRD and SEM-EDX and found that this sample contained quartz and aluminosilicates with interlayer cations such as iron.^93^ The presence of iron can be the reason for the observed fluorescence of AZTD that is observed in the Raman spectrum shown in Table S7 in ESI,† consistent with the previous discussion on the source of for fluorescence in clay minerals.^84,86^

**Fig. 5:**
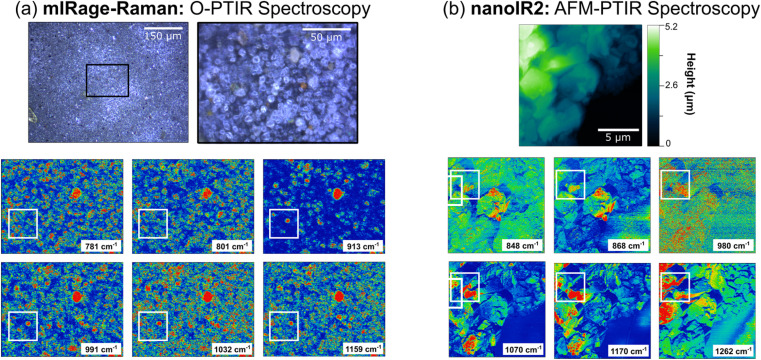
Comparison of spatial heterogeneity detected in Arizona Test Dust (AZTD) on micro- and nanoscales: (a) mIRage-Raman (O-PTIR spectroscopy) and (b) nanoIR2 (AFM-IR spectroscopy), respectively. The optical images are shown at both 10× and 40× magnification as identified by the black box region in the 10× image. The chemical map images are at 10× magnification of a 125 μm × 165 μm region. The AFM 2D height image of an AZTD thin film with spectral maps are shown for a 15 μm × 15 μm region. The boxed areas in white highlight specific regions in which spatial heterogeneities are observed on both the microscale and nanoscale level.

Chemical maps provide additional insights into the heterogeneity in these samples, by spatially resolving the distribution of different species that are present within the sample. In order to generate chemical maps, the QCL laser is tuned to specific wavelengths resonant with peaks in the spectra. Chemical maps that were collected on the microscale and nanoscale using O-PTIR and AFM-IR spectroscopies are shown in Fig. 5. The images and spectral maps collected with O-PTIR spectroscopy are 125 μm × 165 μm whereas for AFM-IR spectroscopy, they are 15 μm × 15 μm. In addition, specific wavenumbers chosen for these spectral maps are based on the spectra that were obtained in Fig. 4. With AZTD, there are two main regions that are distinct – a sharp peak below 868 cm^−1^, representing in-plane bending modes of carbonates, and a more complex region around 910 to 1265 cm^−1^, representing aluminosilicate derivatives.^69,94^

Fig. 5a shows six chemical maps at different wavenumbers collected for a specific region of an AZTD thin film with the mIRage-Raman. O-PTIR maps look somewhat similar, however upon careful inspection, microscopic differences can be observed. Differences observed in the maps shown in the white boxes provide additional information. The 781 cm^−1^ and 801 cm^−1^ O-PTIR maps differ from the rest of the chemical maps, which show a highly IR active circular particle near the center of the white boxes. This suggests that the particle contains components that are IR active at 913 cm^−1^, 991 cm^−1^, 1032 cm^−1^, and 1159 cm^−1^, which can be assigned to aluminosilicates, consistent with the O-PTIR spectra shown in Fig. 4. Moreover, the red-colored hot spots that are specific to the 781 cm^−1^ and 801 cm^−1^ suggest regions in which iron or aluminum oxide could be enriched, as these are close to the frequencies reported in Table S2 in ESI.†Fig. 5b shows AFM-IR chemical maps, where nanoscale heterogeneity are highlighted in the white boxed areas. The 848 cm^−1^ and 868 cm^−1^ maps exhibit similar features that are significantly distinct from the maps at 980 cm^−1^, 1070 cm^−1^, 1100 cm^−1^, and 1170 cm^−1^. However, these maps show differences as well within different regions of the sample, providing insights into the different ratios of species present. Using the information from Fig. S1–S3 in ESI,† the 980 cm^−1^ and 1070 cm^−1^ maps show where regions of either pure aluminum oxide or silicon dioxide, as well as aluminosilicates such as montmorillonite or zeolite, exist. In addition, the maps at 1100 cm^−1^ and 1170 cm^−1^ show where other components, such as sodium sulfate, are present as well. These spectral maps of the different features provide detailed evidence of the heterogeneity of this sample due to the different components present within it and are complementary to the heterogeneity of the spectra shown in Fig. 4. By combining both microscopy and spectroscopy together, along with integrating these different vibrational spectroscopic probes, the heterogeneity of complex materials and the thin films that they form become evident.

## Conclusion

We have investigated geochemical thin films and particles across different length scales from the macro-to the nanoscale using several different vibrational spectroscopic probes. Several important conclusions come from this comparison: (1) the intensities and frequencies for single component mineral thin films differ between the vibrational methods used because of different modes of operation and selection rules; (2) variability in the intensity due to film thickness can be measured on micro and nanoscales; (3) for these geochemical thin films, both single component and the more chemically complex AZTD sample, there is variability in the spectra, intensities and peak positions, for spectra collected on the micro and nanoscale. These variabilities can be due to local chemical environments – including coordination mode, impurities and structural heterogenies as well as different chemical components for AZTD – as well as polarized light interactions with specific crystalline orientations. Amongst the three methods, AFM-IR spectroscopy requires the greatest amount of time for spectral and image data collection but can provide the most detailed information about local environments for thin films and single particles, as well has provided insights on anisotropic interactions with water.^95^ Overall, this study provides an overview of these different vibrational methods for the characterization and analysis of different minerals and geochemical thin films that play a critical role in interfacial geochemistry occurring at the interface of the atmosphere/geosphere and hydrosphere/geosphere.

## Conflicts of interest

There are no conflicts to declare.

## Supplementary Material

RA-013-D3RA05179J-s001
