# Supplementary material for: Vibrational spectroscopy as a probe of heterogeneities within geochemical thin films on macro, micro, and nanoscales
Source: RSC Adv. 2023 Oct 2;13(41):28873–84. doi: 10.1039/d3ra05179j (PMC10543985; doi:10.1039/d3ra05179j)
Supplement: RA-013-D3RA05179J-s001 [file RA-013-D3RA05179J-s001.pdf]

Electronic Supplementary Material (ESI) for RSC Advances.
This journal is © The Royal Society of Chemistry 2023

Electronic Supporting Information:

Vibrational Spectroscopy as a Probe of Geochemical Thin Films and Single Particle on Macro, Micro and Nanoscales

Deborah Kim, Samantha Townsley and Vicki H. Grassian*

Department of Chemistry and Biochemistry, University of California San Diego, La Jolla, CA 92037

*Author to whom correspondence may be addressed. Email: vhgrassian@ucsd.edu

Electronic Supplementary Information Content: This Electronic Supplementary Information (ESI) is 9 pages total with references. The ESI contains 4 figures and 7 tables.

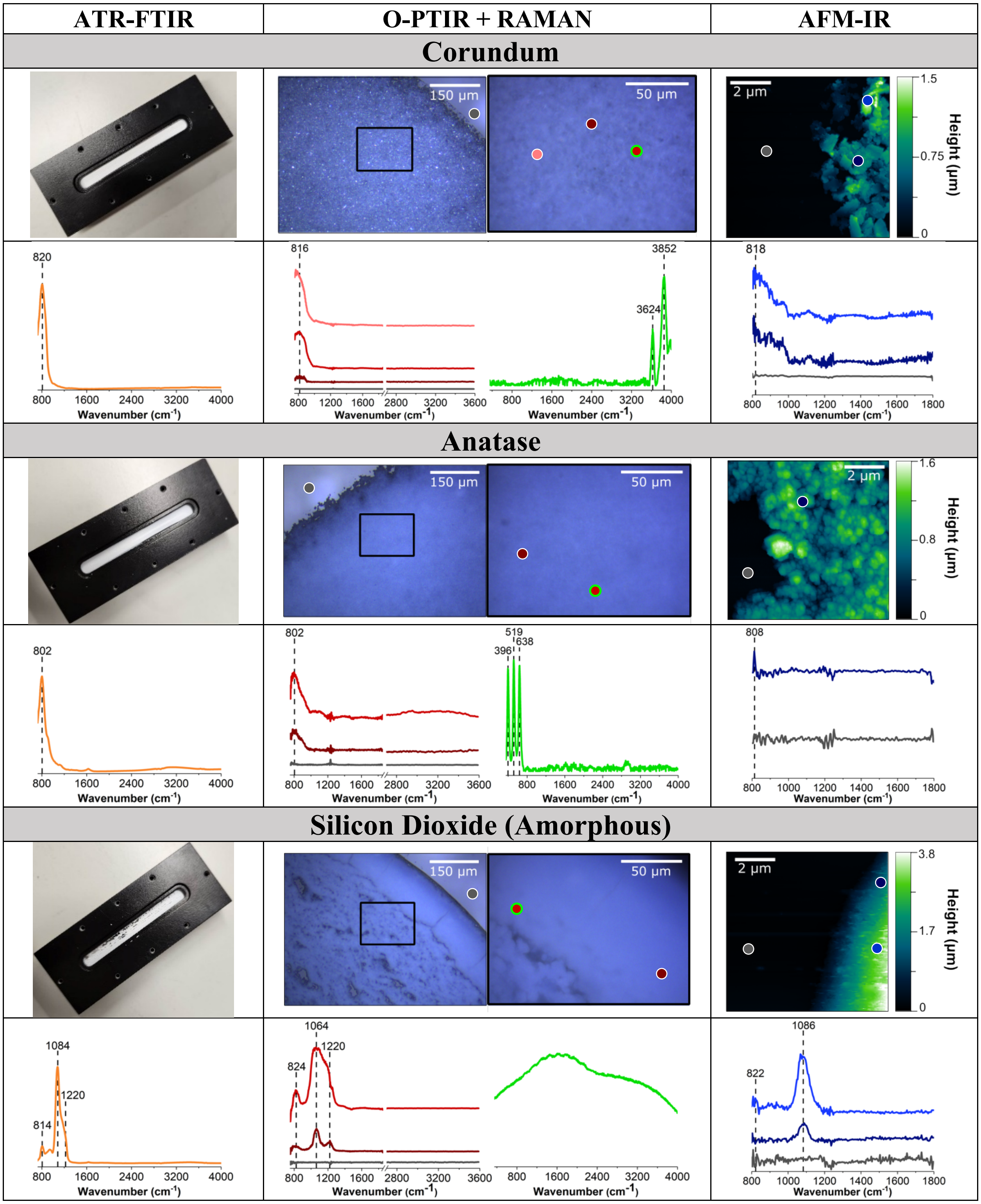

Figure S1. Images and spectra recorded for different oxide minerals – corundum, anatase and silicon dioxide (amorphous) utilizing ATR-FTIR spectroscopy, O-PTIR and Raman spectroscopy and AFM-IR spectroscopy.

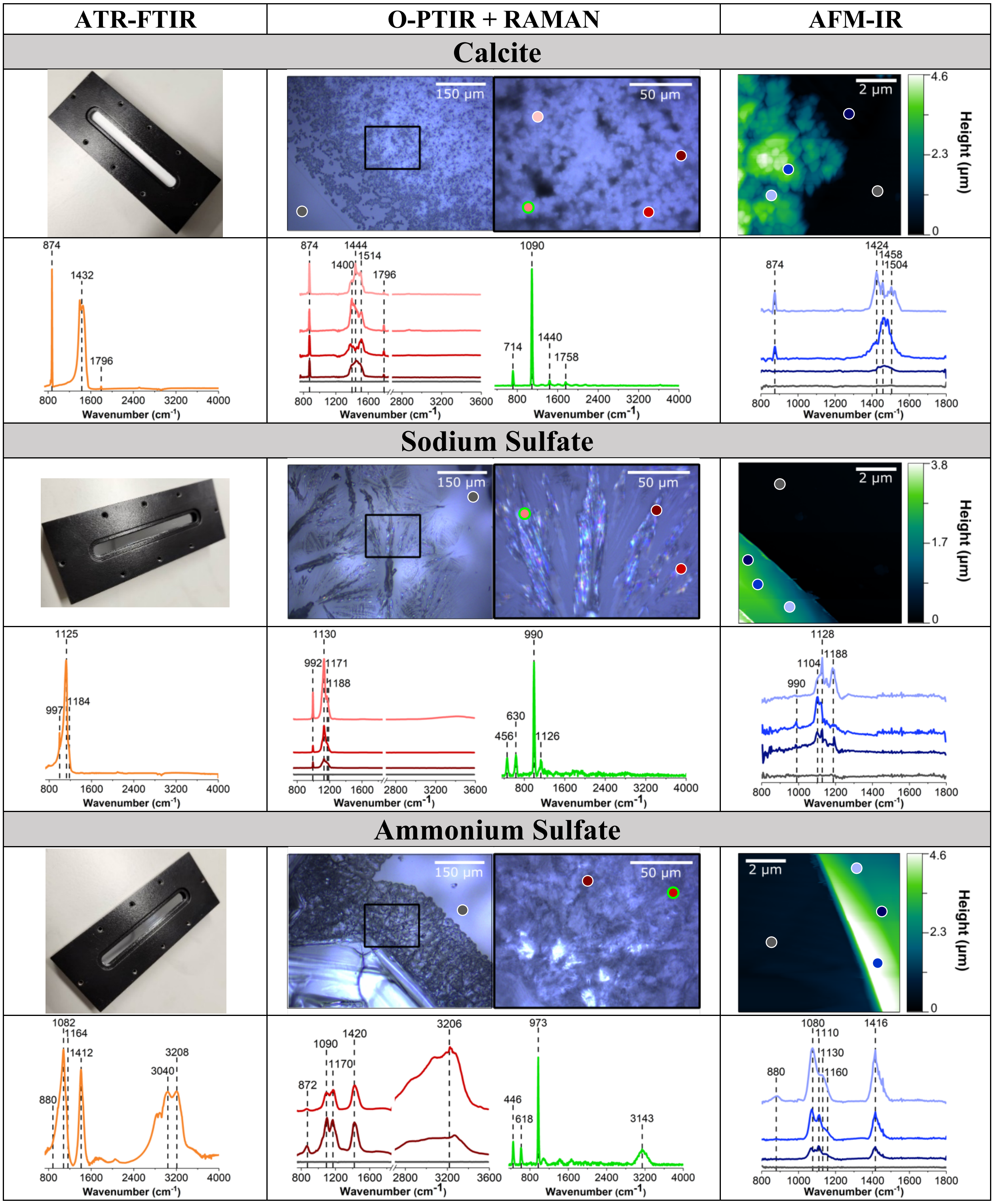

Figure S2. Images and spectra recorded for different carbonate and sulfate minerals – calcite, sodium sulfate and ammonium sulfate utilizing ATR-FTIR spectroscopy, O-PTIR and Raman spectroscopy and AFM-IR spectroscopy.

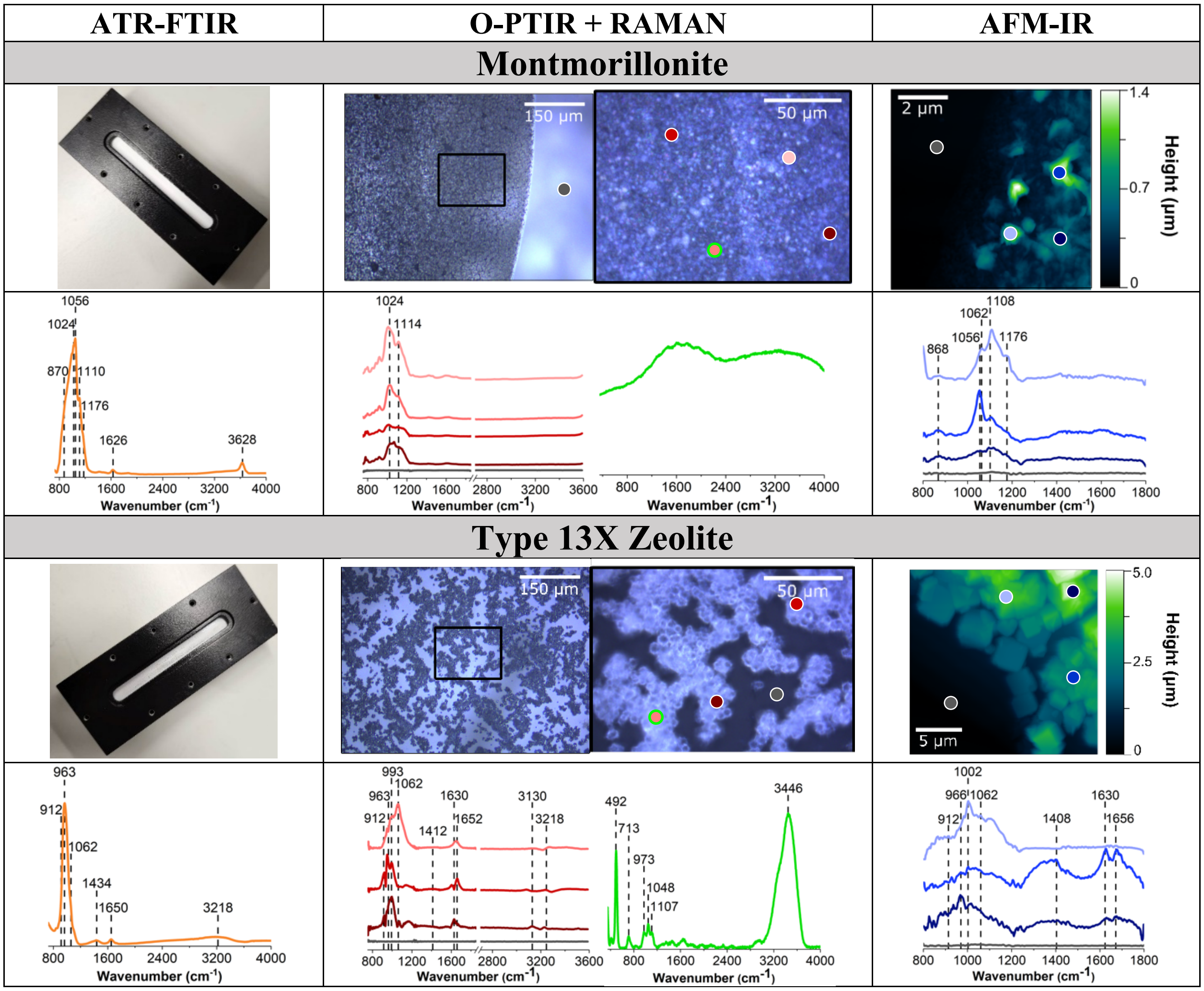

Figure S3. Images and spectra recorded for different aluminosilicates – montmorillonite and zeolite utilizing ATR-FTIR spectroscopy, O-PTIR and Raman spectroscopy and AFM-IR spectroscopy.

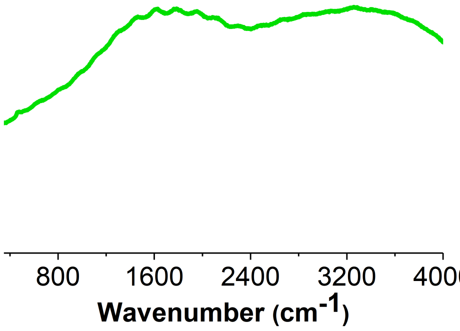

Figure S4. Raman spectrum for Arizona Test Dust (AZTD). As can be seen, fluorescence signal is observed.

Table S1. Summary of classes of minerals, minerals, CAS numbers and sources used.

Classes of Minerals	Minerals	CAS #	Source
Oxides	α-FeOOH (Goethite)	20344-49-4	Alfa Aesar
	α-Al_2O_3 (Corundum)	1344-28-1	Alfa Aesar
	TiO_2 (Anatase)	13463-67-7	Nanostructured & Amorphous Materials Inc.
	SiO_2 (Amorphous)	7631-86-9	Aldrich Chemistry
Carbonates, Sulfates, and Nitrates	$NaNO_3$	7631-99-4	Sigma Aldrich
	$CaCO_3$ (Calcite)	471-34-1	Alfa Aesar
	Na_2SO_4	7757-82-6	Fisher Chemical
	$(NH_4)_2SO_4$	7783-20-2	Fisher Scientific
Clays and Aluminosilicates	Kaolinite	1318-74-7	Sigma-Aldrich
	Montmorillonite	SWy-2 *	The Clay Minerals Society
	Zeolite (Type 13X)	1318-02-1	Sigma Aldrich
Complex Multi-component Samples	Arizona Test Dust	ISO 12103-1*	Powder Technology Inc.

*CAS # is not provided, so specific sample type is provided instead.

Table S2. Vibrational mode assignments of different mineral oxides from infrared spectroscopy utilizing ATR-FTIR, O-PTIR, and AFM-IR spectroscopy.

Sample	Wavenumber (cm^{-1})			Assignment
	ATR-FTIR	*O-PTIR*	*AFM-IR*	
Corundum (α-Al_2O_3)[1]	820	816	818	Al-O stretch
Anatase (TiO_2)[2]	802	802	808	Ti-O stretch
Amorphous SiO_2[3,4]	814	824	822	Si-O stretch
	1084, 1220	1064, 1220	1086	Si-O-Si stretch

Table S3. Vibrational mode assignments of different mineral oxides from micro-Raman spectroscopy.

Sample	Wavenumber (cm^{-1})	Assignment
	Raman	
Corundum (α-Al_2O_3)[5]	3624	O-H stretch
	3852	
Anatase (TiO_2)[6–8]	396	O-Ti-O bend
	519	Ti-O stretch
	638	Ti-O bend
Amorphous SiO_2	Fluorescence signal observed	–

Table S4. Vibrational mode assignments of different carbonate and sulfates from infrared spectroscopy utilizing ATR-FTIR, O-PTIR, and AFM-IR spectroscopy.

Compound	Wavenumber (cm^{-1})			Assignment
	ATR-FTIR	*O-PTIR*	*AFM-IR*	
Calcite ($CaCO_3$)[9,10]	874	874	874	υ_2, Out-of-plane bend
	1432	1400, 1444, 1514	1424, 1458, 1504	υ_3, Asymmetric stretch
	1796	1796	--	Combination bands ($\upsilon_1 + \upsilon_4$)
Sodium Sulfate[11,12]	997	992	990	υ_1, SO_4^{2-} stretch
	1125, 1184	1130, 1171, 1188	1104, 1128, 1188	υ_3, SO_4^{2-} stretch
Ammonium Sulfate[13,14]	1082, 1164	1090, 1170	1080, 1110, 1130, 1160	υ_3, SO_4^{2-} stretch
	1412	1420	1416	υ_4, NH_4^+ stretch
	3040, 3208	3206	--	υ_3, NH_4^+ stretch

Table S5. Vibrational mode assignments of different carbonate and sulfates from micro-Raman spectroscopy.

Compound	Wavenumber (cm^{-1})	Assignment
	Raman	
Calcite ($CaCO_3$)[15]	714	υ_4, in-plane bend
	1090	υ_1, symmetric stretch
	1440	υ_3, asymmetric stretch
	1758	Combination bands ($\upsilon_1 + \upsilon_4$)
Sodium Sulfate[16,17]	456	υ_2, SO_4^{2-} stretch
	630	υ_4, SO_4^{2-} stretch
	990	υ_1, SO_4^{2-} stretch
	1126	υ_3, SO_4^{2-} stretch
Ammonium Sulfate[13,16–19]	446	υ_2, SO_4^{2-} stretch
	618	υ_4, SO_4^{2-} stretch
	973	υ_1, SO_4^{2-} stretch
	3143	N-H stretch

Table S6. Vibrational mode assignments of different aluminosilicates from infrared spectroscopy utilizing ATR-FTIR, O-PTIR, and AFM-IR spectroscopy.

Compound	Wavenumber (cm^{-1})			Assignment
	ATR-FTIR	*O-PTIR*	*AFM-IR*	
Montmorillonite[20]	870	874	868	Al-Fe-OH bend
	1024, 1056, 1110, 1176	1024, 1114	1056, 1062, 1108, 1176	Si-O stretch
	1626	--	--	O-H bend
	3628	--	--	O-H stretch
Zeolite[21]	912, 963, 1062	912, 963, 983, 1062	912, 966, 1002, 1062	Al-O or Si-O asymmetric stretch
	1434	1412	1408	AlO_4 tetrahedra
	1650	1630, 1650	1630, 1656	O-H bend
	3218	3130, 3218	--	O-H bend

Table S7. Vibrational mode assignments and observation of fluorescence for different aluminosilicates and for AZTD

Compound	Wavenumber (cm^{-1})	Assignment
	Raman	
Montmorillonite	Fluorescence signal observed	–
Zeolite[21–24]	492	Al-O-Al or Si-O-Si stretch
	713	Si-O bend
	973	Al-O-Al stretch, SiO_4 stretch
	1048	Al-O bend, Si-O bend
	1107	Si-O stretch
	3446	O-H stretch
Arizona Test Dust (AZTD)	Fluorescence signal observed	–

References.

1 B. Ludwig and T. T. Burke, *Powders*, 2022, **1**, 47–61.

2 M. Al-Amin, S. Chandra Dey, T. U. Rashid, M. Ashaduzzaman and S. M. Shamsuddin, *Int. J. Latest Res. Engineering and Tech.*, 2016, **2**, 14–21.

3 L. A. Zemnukhova, A. E. Panasenko, A. P. Artem'yanov and E. A. Tsoy, *Bioresources*, 2015, **10**, 3713–3723.

4 M. Ocaiñia, V. Forniés and C. J. Serna, *J. Non-Cryst. Sol.,* 1989, **107**, 187-192.

5 S. V. Goryainov, A. S. Krylov, O. P. Polyansky and A. N. Vtyurin, *J. of Raman Spec.*, 2017, **48**, 1431–1437.

6 O. Frank, M. Zukalova, B. Laskova, J. Kürti, J. Koltai and L. Kavan, *Phys. Chem. Chemical Physics*, 2012, **14**, 14567–14572.

7 T. Ohsaka and Y. Fujiki, *J. Ram. Spec.* 1978, **7**, 321-324.

8 Y. Hu, H.-L. Tsai and C.-L. Huang, *J. Euro. Ceram. Soc.* 2003, **23**, 691-696.

9 H. A. Al-Hosney and V. H. Grassian, *Phys. Chem. Chemical Physics*, 2005, **7**, 1266–1276.

10 H. A. Al-Abadleh, H. A. Al-Hosney and V. H. Grassian, in *J. of Mol. Catalysis A: Chem.*, 2005, **228**, 47–54.

11 D. Peak, R. G. Ford and D. L. Sparks, *J. Colloid Int. Sci.*, 1999, **218**, 289–299.

12 X. Wang, Z. Wang, D. Peak, Y. Tang, X. Feng and M. Zhu, *ACS Earth Space Chem.*, 2018, **2**, 387–398.

13 A. L. Bondy, R. M. Kirpes, R. L. Merzel, K. A. Pratt, M. M. Banaszak Holl and A. P. Ault, *Anal. Chem.*, 2017, **89**, 8594–8598.

14 M. C. Yeung, A. K. Y. Lee and C. K. Chan, *Aerosol Sci. and Tech.*, 2009, **43**, 387–399.

15 S. Gunasekaran, G. Anbalagan and S. Pandi, *J. of Raman Spec.*, 2006, **37**, 892–899.

16 M. Jariwala, J. Crawford and D. J. LeCaptain, *Ind. Eng. Chem. Res.*, 2007, **46**, 4900–4905.

17 J. Qiu, X. Li and X. Qi, *J. IEEE Photonics* 2019, **11**, 1-13.

18 J. D. Rindelaub, R. L. Craig, L. Nandy, A. L. Bondy, C. S. Dutcher, P. B. Shepson and A. P. Ault, *J. of Phys. Chem. A*, 2016, **120**, 911–917.

19 M. N. Chan, A. K. Y. Lee and C. K. Chan, *Environ. Sci. Technol.*, 2006, **40**, 6983–6989.

20 H. A. Patel, R. S. Somani, H. C. Bajaj and R. V. Jasra, *Bulletin of Materials Sci.*, 2006, **29**, 133–145.

21 K. Byrappa and B. V Suresh Kumar, *Asian J. Chem.* 2007, **19**, 4933-4935.

22 M. Ritz, L. Vaculíková, J. Kupková, E. Plevová and L. Bartoňová, *Vib. Spectrosc.*, 2016, **84**, 7–15.

23 A. C. Gujar, A. A. Moye, P. A. Coghill, D. C. Teeters, K. P. Roberts and G. L. Price, *Microporous and Mesoporous Mat.*, 2005, **78**, 131–137.

24 Y. L. Tsai, E. Huang, Y. H. Li, H. T. Hung, J. H. Jiang, T. C. Liu, J. N. Fang and H. F. Chen, *Minerals*, 2021, **11**, 1–14.
